# More sense of self-discipline, less procrastination: the mediation of autonomous motivation

**DOI:** 10.3389/fpsyg.2023.1268614

**Published:** 2023-11-23

**Authors:** Su Tao, Yuan Jing

**Affiliations:** ^1^School of Marxism, China University of Geosciences (Beijing), Beijing, China; ^2^School of Humanity and Law, Yanching Institute of Technology, Langfang, Hebei, China

**Keywords:** procrastination, self-discipline, sense of self-discipline, autonomous motivation, self-determination theory

## Abstract

Procrastination is considered a result of failed self-regulation. However, could experiencing a sense of successful self-discipline help to boost motivation and reduce procrastination? To explore this question, two studies were conducted to investigate the relationship between the sense of self-discipline, autonomous motivation, and procrastination. Results showed that trait sense of self-discipline negatively predicted general procrastination (Study 1); self-discipline primed participants procrastinated less than the control group (Study 2); autonomous motivation mediated the relationship between sense of self-discipline and procrastination (Study 1 and Study 2). These findings suggest that cultivating a sense of self-discipline can have positive effects on both autonomous motivation and procrastination, and provide useful guidance for interventions aimed at reducing procrastination.

## 1 Introduction

Procrastination can be defined as the postponement of an intended course of action, despite knowing that such behavior will result in adverse outcomes and discomfort for the individual (Steel, 2007). It is considered an irrational delay (Steel, 2010) and is common characterized by being unnecessary, undesired, and disadvantageous (Svartdal and Steel, 2017). Studies have consistently shown that chronic procrastination not only wastes time but also has negative associations with academic performance (Michinov et al., 2011), counterproductive work behavior (Metin et al., 2016; Jahanzeb et al., 2023), negative work outcomes (O'Neill et al., 2014a,b) and can induce negative emotions that harm well-being (Capan, 2010).

Previous research has found that procrastination is negatively correlated with self-control (Steel, 2007) and self-regulation (Ferrari, 2001; Valenzuela et al., 2020), indicating that individuals who lack self-control and fail to self-regulate tend to procrastinate more. Conversely, if individuals are made to feel that they have successfully exercised self-control and self-regulation in some way, could this feeling lead to a reduction in procrastination? The current study aims to test the hypothesis that experiencing a sense of successful self-discipline may reduce procrastination. Additionally, drawing upon Self-Determination Theory (SDT), we hypothesize that this process is mediated by autonomous motivation.

Self-discipline refers to the individual’s conscious self-restraint to control one’s behavior, speech, or adherence to rules without supervision (Duckworth and Seligman, 2005). In some studies, self-discipline is considered as a facet of conscientiousness of the big-five personality (Lievens et al., 2009; Poropat, 2009). In early research, the measurement of self-discipline included various aspects, such as self-control, impulsiveness personality, and delay gratification (Duckworth and Seligman, 2005, 2006). Although self-control and self-discipline share some common elements, research shows that they differ in measurement structure (Hagger et al., 2021). Self-control focuses on regulating behavior and making choices, while self-discipline emphasizes regularity and restraint, which is the feeling of being able to successfully exercise self-control when compelled to act (Zimmerman and Kitsantas, 2014). Some specialized Self-Discipline Scales have also emerged, encompassing not only aspects of self-control but also elements like responsibility, conscious effort, willpower, and achieving goals (Jung et al., 2017; Simsir and Dilmac, 2022).

Sense of self-discipline, essentially, is the subjective awareness and feelings of self-discipline. Generally, individuals experience a sense of self-discipline in two ways: by doing what they should do but do not want to do (e.g., brushing their teeth before bed when tired), or by not doing what they want to do but should not do (e.g., resisting distractions during class) (Zimmerman and Kitsantas, 2014). Sense of self-discipline, self-efficacy, and sense of control all refer to experiencing a sense of control over oneself, but they have distinct differences. Sense of self-discipline is related to the awareness and feeling of self-restraint, while self-efficacy refers to the expectation of being able to complete a task (Bandura, 1977). Sense of control emphasizes control over the external environment rather than self-restraint (Ajzen, 2002).

Based on the findings from previous research, it can be deduced that a sense of successful self-discipline is likely to mitigate procrastination, and this can be comprehended through three key perspectives. Firstly, numerous studies have suggested that self-discipline can bolster performance in tasks that require self-control and is linked to a range of positive outcomes, including academic success, decreased procrastination, and delayed gratification (Duckworth and Seligman, 2005; Waschull, 2005; Duckworth and Seligman, 2006; Shi and Qu, 2022). Secondly, a substantial amount of research indicates that self-efficacy and a sense of control, both closely related to a sense of successful self-discipline, can effectively reduce procrastination (Klassen et al., 2008; Katz et al., 2014; Wäschle et al., 2014; Zhang L. et al., 2018; Zhang Y. et al., 2018; Shao et al., 2023; Tian et al., 2023). Thirdly, heightened feelings of self-discipline can strengthen domains of behavioral self-control, such as promoting healthy eating behavior and pro-social decision-making (Tian et al., 2018). By resisting temptations and practicing self-control, individuals may reduce procrastination.

However, the relationship between sense of self-discipline and procrastination may also be the opposite. On one hand, according to the Strength Model of Self-Control (Baumeister et al., 1998, 2007), self-control resources are limited, and successful self-discipline depletes these resources, thereby reducing one’s subsequent ability to exert self-control, even on unrelated tasks (Hagger et al., 2010). On the other hand, Ariely and Wertenbroch (2002) discovered in their research that when individuals become aware of their self-control issues, they may tend to set a deadline to manage their procrastination, which may decrease the procrastination caused by a lack of self-discipline. In light of these two lines of evidence, the authors posits that within the Strength Model of Self-Control, although efforts in self-discipline deplete resources, the positive outcomes, such as emotions, brought about by successful self-discipline may potentially serve as a supplement to psychological resources (Fredrickson, 2001), thereby contributing to subsequent performance. While the deadline-setting strategy proposed in the latter view may only attempt to control procrastination but may not necessarily be effective.

Given this debate, this study assumes that the former result holds in a broader context: when individuals feel self-disciplined, it enhances their ability to complete tasks and thereby reduces procrastination. Accordingly, the following hypotheses are proposed in this study:

Hypothesis 1: Trait sense of self-discipline is negatively related to general procrastination.

Hypothesis 2: Priming sense of self-discipline reduces situational procrastination.

How does the sense of self-discipline influence procrastination? This study looks at this relationship from the perspective of Self-Determination Theory (SDT) and suggests that the sense of self-discipline reduces procrastination by increasing the level of autonomous motivation. Autonomous motivation refers to a combination of intrinsic motives on the motivation continuum, emphasizing the individual’s choice to engage in a particular behavior based on their interests or personal beliefs, or to act according to their own will and choices (Vansteenkiste and Sheldon, 2006; Ratelle et al., 2007). The relationship between the sense of self-discipline and autonomous motivation can be understood in three distinct ways.

Firstly, feeling self-disciplined can fulfill people’s basic psychological needs and lead to a stronger sense of autonomous motivation. According to SDT, humans have three basic psychological needs: autonomy, competence, and relatedness (Ryan and Deci, 2020). When individuals engage in tasks that offer minimal constraints and some choice, it can meet people’s needs for autonomy and free will, thereby enhancing their sense of self-discipline (Ryan and Connell, 1989; Byron and Khazanchi, 2012). Such experiences can lead to a more self-directed and self-motivated approach to task completion, thereby reducing the tendency to procrastinate (Soenens and Vansteenkiste, 2010). Self-discipline is not a behavior adopted under environmental or peer pressure, but rather a behavior taken before being forced. Therefore, the core of the sense of self-discipline is that the behavior comes from personal will rather than external monitoring, and it is the individual’s active choice to do what is necessary. People who feel self-disciplined can fulfill their need for autonomy, and thus it can be expected that they will have higher autonomous motivation. Furthermore, because the sense of self-discipline brings about a sense of self-worth (Ryan and Connell, 1989; Gagne et al., 2015) and competence (Deci and Ryan, 2000; Byron and Khazanchi, 2012), it further promotes identity and integration regulation in autonomous motivation.

Secondly, feeling self-disciplined can be directly associated with autonomous motivation. Self-discipline often manifests itself in tasks that individuals are reluctant to do or are lazy about but must complete. In such cases, individuals must overcome distractions and resistance to shift from a passive to an active state. Research has shown that self-discipline can enhance an individual’s sense of initiative (Zimmerman and Kitsantas, 2014). Feeling self-disciplined fosters strong willpower and courage, resulting in a sense of inner peace (Simsir and Dilmac, 2022), as well as a heightened sense of focus and alertness (Tian et al., 2018). All these factors collectively contribute to an individual’s autonomous motivation.

Thirdly, although motivation is typically viewed as a predictor of behavior, Self-perception theory suggests that individuals often make inferences about their attitudes, emotions, and other internal states by observing their behavior (Bem, 1967; Ariely and Norton, 2011). By recognizing that they have successfully exercised self-restraint and self-control in some way, individuals may further reinforce their autonomous motivation and develop a stronger interest in tasks they were previously unwilling to do. Previous studies have also demonstrated the significance of improving individuals’ sense of self-discipline in maintaining autonomous motivation as an internal mechanism for academic success (Pintrich, 2004; Nishimura and Sakurai, 2013; Orsini et al., 2019; Sarkis et al., 2020). Therefore, this study hypothesizes that the sense of self-discipline can positively predict an individual’s autonomous motivation.

Autonomous motivation has consistently shown a negative correlation with procrastination, as evidenced by several studies. For example, Brownlow and Reasinger (2000) found that higher levels of autonomous motivation were associated with less procrastination. Additionally, Vansteenkiste et al. (2010) found that autonomous regulation mediated the positive relationship between adaptive perfectionism and learning outcomes. Studies have also shown that individuals with higher achievement motivation tend to have less procrastination, with the neural basis for this relationship being recently discovered (Li et al., 2022). Based on the above results, this study proposes:

Hypothesis 3: the relationship between sense of self-discipline and procrastination is mediated by autonomous motivation.

**Figure 1:**
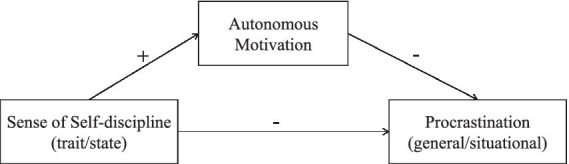
Diagram of hypothesized relationships between variables.

The proposed model for the study is shown in Figure 1.

In summary, the perspective of self-determination theory is used to examine the relationship between the sense of self-discipline, autonomous motivation, and procrastination in this study. The hypothesis is that a higher sense of self-discipline leads to higher levels of autonomous motivation, which in turn reduces procrastination. Study 1 conducted correlation analysis and mediation tests to examine the relationship between trait sense of self-discipline, autonomous motivation, and general procrastination. Building on these findings, Study 2 further investigates whether priming a sense of self-discipline can increase an individual’s autonomous motivation, ultimately leading to a reduction in situational procrastination.

## 2 Study 1: the effect of trait sense of self-discipline and autonomous motivation on general procrastination

Since self-discipline can be viewed as an individual trait, the sense of self-discipline is relatively stable. Duckworth and Seligman (2005) have argued that self-discipline should be measured in a quite broad way. However, in this study, we are not concerned about the individual’s capability of self-discipline. Instead, our focus is on examining individuals’ perceptions of self-discipline. Therefore, a self-report measurement is utilized. In Study 1, questionnaires were used to explore the correlation between stable self-discipline perception, autonomous motivation, and general procrastination. The study tested Hypothesis 1 and 2, that is, trait sense of self-discipline is negatively related to general procrastination; the relationship between sense of self-discipline and procrastination is mediated by autonomous motivation.

### 2.1 Methods

#### 2.1.1 Participants

The sample consisted of 381 college students. After removing the questionnaires with repeated answers or some items missing, 377 valid participants were included, 207 males and 170 females, *M*_age_ = 19.82 years, *SD* = 1.48.

#### 2.1.2 Measures

##### 2.1.2.1 Procrastination

The General Procrastination Scale (GPS), developed by Lay (1986), was used to assess behaviors and emotions associated with procrastination in both daily activities and academic settings. The Chinese version scale consists of 13 items, with participants rating their responses on a 5-point scale ranging from 1 (completely inconsistent) to 5 (completely consistent). Higher scores on the GPS indicate more severe procrastination tendencies. The *Cronbach’s α* in this study was 0.810.

##### 2.1.2.2 Autonomous motivation

This study employed the Chinese version of the Autonomous Motivation Scale (Jia, 2016) to measure autonomous motivation. This scale was adapted from the Motivation at Work Scale (MAWS) (Gagné et al., 2010) and Academic Self-regulation Questionnaire (SRQ-A) (Ryan and Connell, 1989), addressing the limitation of these two scales in measuring motivation in a single domain. After revision, the scale demonstrates good reliability and validity, and can be used to measure individuals’ general autonomous willingness, identification, and interest in completing academic and daily tasks. The scale consists of 14 items, such as “When I do something in my daily life or studies, it is generally because I enjoy doing it” and “When I do something in my daily life or studies, it is generally because it aligns with my life goals.” Participants rate their responses on a 5-point scale, ranging from 1 (completely inconsistent) to 5 (completely consistent). Higher scores on the scale indicate higher levels of autonomous motivation. The Cronbach’s alpha coefficient in this study was 0.797.

##### 2.1.2.3 Sense of self-discipline

The Sense of Self-Discipline test adapted from Tian et al. (2018) consisted of four items: “I felt self-disciplined,” “I felt like my willpower was gone” (reverse-scored), “I felt mentally strong,” and “I felt sharp and focused.” Participants rated the frequency with which they experienced these feelings in their daily lives on a scale from 1 (never) to 7 (often). Higher scores on the scale indicate a higher level of trait sense of self-discipline. The *Cronbach’s α* in this study was 0.788.

### 2.2 Results

To explore the relationship between general procrastination, autonomous motivation, and sense of self-discipline, a correlation analysis was conducted. The results revealed that procrastination was significantly negatively correlated with both autonomous motivation (*r* = −0.24, *p* < 0.001) and sense of self-discipline (*r* = −0.23, *p* < 0.001). Furthermore, autonomous motivation and sense of self-discipline were significantly positively correlated (*r* = 0.31, *p* < 0.001).

A multiple linear regression analysis was conducted with trait sense of self-discipline as the predictor variable, general procrastination as the outcome variable, autonomous motivation as a potential mediator, and gender as a covariate. Results are shown in Table 1.

**Table 1:** Regression of sense of self-discipline and autonomous motivation on general procrastination (*N* = 377).

	Dependent variables	Predictor	*β*	*t*	*R* ^2^	*F*
Step1	Procrastination	Sense of self-discipline	−0.23	−4.53***	0.23	20.52***
Step2	Autonomous motivation	Sense of self-discipline	0.31	6.36***	0.31	40.39***
Step3	Procrastination	Sense of self-discipline	−0.17	−3.26***	0.29	16.92***
		Autonomous motivation	−0.19	−3.56***		

***p < 0.001.

Firstly, a regression analysis was conducted with sense of self-discipline as the independent variable and general procrastination as the dependent variable. The results showed that sense of self-discipline significantly negatively predicted general procrastination (*β* = −0.23, *t* = −4.53, *p* < 0.001), supporting Hypothesis 1. Secondly, sense of self-discipline significantly predicted the candidate mediator variable of autonomous motivation (*β* = 0.31, *t* = 6.36, *p* < 0.001). Thirdly, after including autonomous motivation as a mediator variable, the predictive effect of sense of self-discipline on general procrastination was reduced but still significant (*β* = −0.17, *t* = −3.26, *p* = 0.001). The negative predictive effect of autonomous motivation on general procrastination was also significant (*β* = −0.19, *t* = −3.56, *p* < 0.001). Bootstrap tests with bias correction showed that the mediating effect of autonomous motivation was significant, with an effect value of −0.10 and a *95% CI* of [−0.19, −0.04]. The mediating effect (−0.10) accounted for 25.64% of the total effect (−0.39), indicating that autonomous motivation played a partial mediating role between sense of self-discipline and general procrastination, supporting Hypothesis 3.

## 3 Study 2: activating sense of self-discipline reduces situational procrastination

In Study 1, questionnaires were administered to explore the associations between variables, and results confirmed Hypothesis 1, which states that trait sense of self-discipline is a negative predictor of general procrastination, as well as Hypothesis 3, which suggests that autonomous motivation plays a partial mediating role in the relationship between sense of self-discipline and general procrastination. Additionally, sense of self-discipline can also be a short-term feeling that can be activated through some methods. Can this state sense of self-discipline enhance autonomous motivation and reduce procrastination? Study 2 conducted an experimental study to address this question by activating participants’ sense of self-discipline by recalling self-disciplined events.

To distinguish from the general measurement of procrastination, the present study developed a task-based measure of procrastination. Previous studies have shown that chronic procrastinators spend more time on interesting alternative tasks rather than evaluative tasks (Ferrari and Tice, 2000; Svartdal and Lokke, 2022). When people prepare for a series of academic or work tasks of different difficulties, choosing to relax or start with the easiest task and then complete the difficult ones later when they have to be completed can be considered as procrastination. Conversely, if people do not make choices about the tasks and can immediately start working on each task, even the most difficult and time-consuming ones, it means they have refused procrastination. Therefore, this study used three exam questions and participants’ answer order for questions of different difficulty levels as the indicator of procrastination. This measurement method was used to test Hypothesis 2 and 3, that is, priming sense of self-discipline reduces situational procrastination, and the relationship between sense of self-discipline and procrastination is mediated by autonomous motivation.

### 3.1 Methods

#### 3.1.1 Participants

The sample included 97 college students (*M*_age_ = 20.33 years, *SD* = 2.02), 51 males, and 46 females. Participants were randomly assigned to different groups, including 52 in the sense of self-discipline priming group and 45 in the control group.

#### 3.1.2 Measures

##### 3.1.2.1 Procrastination behavior

This study measured procrastination using three translation questions of different levels of difficulty. Twenty college students were recruited for a pilot study and asked to translate 14 English sentences of similar length but different levels of difficulty into Chinese, and rate the difficulty from 1 (very easy) to 5 (very difficult). Based on the participants’ ratings, three translation questions were selected for the procrastination test in this study: the most difficult (*M* = 3.70 ± 0.19), the moderate difficulty (*M* = 2.50 ± 0.13), and the easiest (*M* = 1.60 ± 0.19) task. The three tasks were: “Insurance must be covered when the goods are exported in case they are lost or damaged in transit. (Hard),” “If we do not receive payment by the end of this month, we have no choice but to take legal action. (moderate),” and “If you watch the sky for an hour after the sun goes down, you may see some stars moving. (Easy).” In a class, students were told that their scores on these tasks would count as part of their regular assignments, and the three tasks were arranged in order of difficulty from hardest to easiest, with scores of 5, 3, and 2, respectively. Participants were asked to complete the tasks in the assigned order as much as possible. During the test, the actual order of completion was recorded as an indicator of procrastination. The score was calculated based on the difference between the participant’s actual order of completion and the assigned order. For example, if the participant completed the hard task (Task 1) after the moderate task (Task 2), one point was deducted, and so on. The score ranged from 0 to 4, with higher scores indicating a stronger tendency to avoid difficult tasks and complete easier tasks despite knowing that the difficult tasks carry higher scores, which reflects stronger procrastination. The procrastination score was treated as a continuous variable for subsequent statistical analysis.

To examine the validity of the self-developed procrastination behavior measurement, we conducted a preliminary study by comparing the procrastination indicator with two established questionnaires. The first questionnaire was the Irrational Procrastination Scale (IPS, 9 items) (Steel, 2010) focusing on implemental delay. The second one was the Procrastination Assessment Scale for Students (PASS, section one, 12 items) (Solomon and Rothblum, 1984), which is widely used to assess academic procrastination. A total of 139 university students participated in the study (*M*_age_ = 19.40 ± 1.78). The results showed that the procrastination indicator correlated with IPS at 0.30, *p* < 0.001, and with PASS at 0.36, *p* < 0.001. This suggests that the procrastination indicator used in this study effectively measures individuals’ procrastination tendencies.

##### 3.1.2.2 Autonomous motivation

We used the Autonomous Motivation Scale from Study 1, but it was adapted to measure the motivation of participants in completing this translation test.

##### 3.1.2.3 Sense of self-discipline

Measurement of sense of self-discipline was similar to that in Study 1, but adapted to measure the sense of self-discipline when participants completed this translation test.

#### 3.1.3 Design and procedure

This study is a single-factor between-participants design, in which participants were randomly assigned to either the sense of self-discipline priming group or the control group. Each participant was informed that they would complete several translation tasks and evaluate their subjective feelings while doing so.

The sense of self-discipline priming was implemented first, in which participants were asked to recall an experience in which they completed a task without supervision through self-discipline, recall as many details and feelings as possible, and write them down. The events written down by participants include making a study plan and completing it, practicing an instrument, dieting, exercising, and so on. To match the control group with the self-discipline priming group, participants in the control group were asked to recall an unrelated event, i.e., any event that happened yesterday, and recall as many details and feelings as possible.

After completing the recall, participants immediately answered the translation questions, followed by completing a questionnaire on their level of sense of self-discipline and autonomous motivation, and the experimenter recorded the order in which participants translated the three sentences.

### 3.2 Results

First, a manipulation check was conducted on the sense of self-discipline of the priming group and the control group. The results showed a significant difference in the sense of self-discipline questionnaire scores between the priming group (*M* = 5.76 ± 0.81) and the control group (*M* = 5.03 ± 0.73), *t* = 4.618, *p* < 0.001, *df* = 95. This indicates that recalling experiences can enhance participants’ sense of self-discipline and that the priming was effective.

An independent samples t-test was conducted with the sense of self-discipline priming as the independent variable and procrastination as the dependent variable. The results indicated that the procrastination score of the self-discipline initiation group (*M* = 1.37 ± 1.44) was significantly lower than that of the control group (*M* = 2.07 ± 1.45), *t* = −2.381, *p* = 0.019, *df* = 95, suggesting that enhancing state sense of self-discipline can reduce procrastination, supporting Hypothesis 2.

To examine the relationships among sense of self-discipline, autonomous motivation, and procrastination, regression analyses were conducted with gender as a covariate. The results are presented in Table 2. As shown in the table, priming of sense of self-discipline had a significant effect on sense of self-discipline (*β* = 0.43, *t* = 4.62, *p* < 0.001). When both sense of self-discipline and the priming were included as predictors of autonomous motivation, only sense of self-discipline significantly predicted autonomous motivation (*β* = 0.51, *t* = 5.42, *p* < 0.001), while the priming did not (*β* = 0.12, *t* = 1.32, *p* = 0.19). When all three predictors (the priming, sense of self-discipline, and autonomous motivation) were included in the regression model to predict procrastination, only autonomous motivation significantly predicted procrastination (*β* = −0.25, *t* = −2.11, *p* = 0.04).

**Table 2:** Regression of self-discipline priming, sense of self-discipline, and autonomous motivation on situational procrastination (*N* = 97).

	Dependent variables	Predictor	*β*	*t*	*p*	*R^2^*	*F*
Step1	Sense of self-discipline	Self-discipline priming	0.43	4.62	<0.001	0.18	21.32***
Step2	Autonomous motivation	Self-discipline priming	0.12	1.32	0.19	0.33	22.83***
		Sense of self-discipline	0.51	5.42	<0.001		
Step3	Procrastination	Self-discipline priming	−0.15	−1.38	0.17	0.11	3.93*
		Sense of self-discipline	0.00	−0.01	0.99		
		Autonomous motivation	−0.25	−2.11	0.04		

***p < 0.001, *p < 0.05.

To test the mediating role of feelings of self-discipline, a chain mediation effect test was conducted using Model 6 in the PROCESS plugin with priming as the independent variable, sense of self-discipline and autonomous motivation as mediators, and procrastination as the dependent variable. The results are shown in Table 3.

**Table 3:** Test of chain mediation effects.

	Effect	BootSE	BootLLCI	BootULCI
Total effect	−0.26	0.15	−0.56	0.06
Priming ➔Sense of self-discipline➔ procrastination	−0.00	0.16	−0.32	0.33
Priming ➔ autonomous motivation ➔ procrastination	−0.09	0.07	−0.27	0.03
Priming ➔sense of self-discipline➔ autonomous motivation ➔ procrastination	−0.16	0.08	−0.37	−0.03

From the table, it can be seen that neither sense of self-discipline nor autonomous motivation alone as mediators are significant in the process of priming predicting procrastination, as the bootstrap *95% CI* for both upper and lower limits contain 0. However, priming had a significant chain mediating effect on procrastination through the sense of self-discipline and autonomous motivation, with a bootstrap *95% CI* of [−0.37, −0.03] not including 0. This result suggests that the sense of self-discipline induced by priming does not have a direct impact on procrastination, nor does priming directly predict autonomous motivation. However, priming enhances the sense of self-discipline, subsequently triggering autonomous motivation, which ultimately leads to a reduction in procrastination. This further strengthens the support for hypothesis 3.

## 4 General discussion

Procrastination is a prevalent problem in contemporary society that wastes valuable time resources and hinders academic and work performance. The chaotic pace of life may cause individuals to feel out of control, leading to lower levels of happiness and life satisfaction. This study aims to find a psychological state that can improve procrastination.

Through two studies, this research examines the impact of both trait and state sense of self-discipline on procrastination and the mediating role of autonomous motivation. Study 1 utilized questionnaires to explore the relationship between trait sense of self-discipline, autonomous motivation, and general procrastination. The results supported H1 and H3, namely that the trait sense of self-discipline negatively predicted general procrastination. Individuals who frequently perceive themselves as self-disciplined are less likely to procrastinate in daily life and academics. This is mainly because they have a stronger autonomous motivation when engaging in these activities, meaning that they see intrinsic value in and are voluntarily choosing to engage in these behaviors. Conversely, individuals with a weaker sense of self-discipline exhibit lower levels of autonomous motivation, making them less willing to initiate tasks, thus leading to procrastination. Study 2 used an experimental design and developed a procrastination behavior test to investigate the influence of state sense of self-discipline on procrastination by recalling self-discipline tasks. The results supported H2 and H3, indicating that participants in the self-discipline priming group had a higher sense of self-discipline and lower procrastination than those in the control group. Further analysis showed a chain mediation effect between self-discipline priming, sense of self-discipline, autonomous motivation, and procrastination. That is, recalling self-discipline tasks improved individuals’ sense of self-discipline, prompting them to have a stronger autonomous motivation to complete the translation test, leading them to tackle the questions in the order of difficulty and showing less procrastination. Conversely, randomly recalling tasks did not improve individuals’ sense of self-discipline, leading to lower interest or volition in completing the translation test and an increased likelihood of prioritizing easier questions and avoiding difficult ones, i.e., procrastination.

Two studies have consistently shown a negative correlation between the sense of self-discipline and procrastination, as well as the mediating role of autonomous motivation. This suggests that not only does one experience a low sense of self-discipline due to their procrastination, but the reverse process is also true: a low sense of self-discipline itself may reduce autonomous motivation and lead to procrastination. In previous research, self-efficacy has been identified as an important factor influencing motivation and procrastination. For instance, procrastination is associated with low academic self-efficacy (Nemtcan et al., 2022), and low self-efficacy can mediate the relationship between low self-esteem and procrastination (Zhang L. et al., 2018; Zhang Y. et al., 2018). Study self-efficacy also mediates the relationship between study habits and procrastination (Svartdal et al., 2022), and can reduce an individual’s expectation of success, damage motivation, and ultimately lead to procrastination (Liu et al., 2020). Improving self-efficacy typically requires experiences of completing specific tasks in a particular field, and procrastination may lead to poor performance in that area, thus lowering self-efficacy. Low self-efficacy can in turn lead to more procrastination, creating a failure spiral that is difficult to break (Steel, 2007). However, unlike self-efficacy, a sense of self-discipline is not solely dependent on completing specific tasks in a particular field, but can also be cultivated by exercising self-discipline in areas of proficiency. This feeling satisfies people’s basic psychological needs for autonomy and competence. By satisfying their psychological needs, individuals can integrate tasks they undertake with personal values, generate curiosity and interest, and experience will and freedom in action (Deci and Ryan, 2000). This acquired intrinsic motivation drives people to actively complete necessary tasks, thereby reducing procrastination.

### 4.1 Insights and implications

Firstly, this study highlights the role of subjective experiences in procrastination. Predictors of procrastination include personality traits, task characteristics, external environments, and demographics (Steel, 2007). Previous studies on the factors affecting procrastination have largely focused on the level of personality traits, especially on the relationship between the five-factor model and procrastination behavior. For example, some studies found that conscientiousness and its facets were the strongest correlates with procrastination among all personality traits (Steel and Klingsieck, 2016), and maladaptive perfectionism was also identified as an important factor affecting procrastination (Fee and Tangney, 2000; Sepiadou and Metallidou, 2022; Zhang et al., 2022). In addition, individuals with low self-esteem are prone to anxiety and negative emotions when facing tasks, especially challenging ones, and are more likely to procrastinate (Ferrari, 2000; Di Fabio, 2006; Steel, 2007). These factors are relatively stable, and while most research explores the mechanisms by which they contribute to procrastination, it can be challenging to develop targeted interventions. This study aims to identify a more easily intervenable situational subjective experience, namely the sense of self-discipline, and investigate how an individual’s perception of their level of self-discipline affects their motivation and, consequently, their tendency to procrastinate.

Secondly, a new method for measuring procrastination is proposed in this study. While existing procrastination measurement methods are mostly self-reported, a study by Svartdal and Steel (2017) examined the psychometric properties of five prevalent procrastination scales. However, questionnaires measure relatively stable procrastination, whereas this study attempts to predict procrastination from a more specific behavioral perspective. The influential method for measuring procrastination at the behavioral level is the one proposed by Ferrari and Tice (2000) and Ferrari (2001), which measures procrastination based on the choice of completing a math task or engaging in other fun activities (e.g., playing a video game or working on a puzzle) when given tasks to complete. However, this method is difficult to operate and is more influenced by the procrastinator’s procrastination level. The measurement method provided in this study is more straightforward, where participants only need to complete a translation task. This method generates a behavioral-level procrastination measurement, characterized by the tendency of participants to prioritize easy tasks even when given instructions and leave difficult tasks until the last minute. This method may be sensitive to situational factors and is suitable for use in experimental designs.

Thirdly, the results of this study make a significant contribution to interventions for procrastination. Scholars have used various psychological counseling therapies such as Cognitive Behavioral Therapy (CBT) and rational emotive behavior therapy (REBT) to effectively reduce procrastination in academic and work settings (Kobori et al., 2020; Moharram-Nejadifard et al., 2020; Ugwuanyi et al., 2020; Balkis and Duru, 2022). Other researchers have pointed out that a lack of appropriate time management strategies is a significant cause of procrastination and have implemented interventions targeting procrastination from a time management perspective, as in van Eerde (2003). Additionally, some scholars have suggested that changing the way tasks are presented, such as through group work, can reduce academic procrastination (Koppenborg et al., 2023). Glick and Orsillo (2015) compared the effectiveness of these interventions and found that acceptance-based behavior therapies (ABBTs) were more effective for chronic procrastinators. While most of these methods require professional and long-term intervention, the approach provided in this study to improve one’s sense of self-discipline is more convenient for individuals to implement in their daily lives. A sense of self-discipline can be obtained not only from completing current tasks but also from simpler methods, such as recalling past self-disciplined events. Studies have shown that setting strict and repetitive ritual behaviors increases the sense of self-discipline. Participants who complete ritual steps have a stronger sense of self-discipline than those who perform random actions (Tian et al., 2018). Even green space can help boys and girls lead more self-disciplined lives (Taylor et al., 2002). These provide valuable strategies for intervening in procrastination and breaking the cycle of procrastination.

### 4.2 Limitations and suggestions for future research

Firstly, it is important to acknowledge that the participants in this study were exclusively college students, which may restrict the generalizability of the results. While academic procrastination is one of the most extensively researched types of procrastination (Zacks and Hen, 2018), it is crucial to investigate other forms and contexts of procrastination. Although the general procrastination scale employed in this study evaluates procrastination in both academic and non-academic areas, future research could involve a more diverse participant pool and include a broader range of situations to enhance the universality of the findings.

Secondly, the study utilized a translation test to measure the behavioral index of procrastination based on the order in which participants answered the questions, starting from the difficult ones to the easy ones. This approach is consistent with the definition of procrastination, as it reflects the tendency to prioritize easier tasks over more challenging ones until the last minute. However, there are two potential issues associated with this methodology. The first concern is that it may not completely eliminate the possibility of response bias, where some participants might prioritize easier questions in order to achieve higher scores. The second concern pertains to the potential impact of participants’ English proficiency on the results. In our study, all participants were enrolled in intermediate-level English classes at a university, which means their English proficiency levels were relatively similar. Nevertheless, it might be necessary to modify this measurement method according to the participants’ individual abilities. To mitigate this limitation, future studies could incorporate additional indicators to measure situational procrastination, which would allow for a more comprehensive assessment of the construct.

Thirdly, the participants were required to complete recall tasks to enhance their sense of self-discipline. Nevertheless, other approaches could be considered to cultivate a sense of self-discipline. For instance, individuals may acquire vicarious experiences of self-discipline through observational learning, such as watching online streaming of people engaged in study or work, which is increasingly popular. Observing these self-disciplinarians may encourage individuals to immerse themselves in learning or work, thus enhancing their subjective sense of self-discipline, which may lead to behavioral changes. Future research endeavors should consider the implementation of diverse methods aimed at improving the sense of self-discipline, thereby making a substantial contribution to procrastination intervention.

## 5 Conclusion

The present study posits that the sense of self-discipline is a predictor of procrastination, and this relationship is mediated by autonomous motivation. Specifically, participants with greater trait or state sense of self-discipline are more likely to exhibit higher levels of autonomous motivation, which in turn leads to reduced tendencies toward procrastination. Furthermore, the findings suggest that interventions aimed at enhancing an individual’s sense of self-discipline may serve as a viable approach to reducing procrastination.

## Data availability statement

The datasets presented in this study can be found in online repositories. The names of the repository/repositories and accession number(s) can be found at: https://pan.baidu.com/s/1CCJKu_fbSK-XuhFoc2t_lA?pwd=wcyv.

## Ethics statement

The studies involving humans were approved by the Institutional Review Board of School of Marxism, China University of Geosciences (Beijing). The studies were conducted in accordance with the local legislation and institutional requirements. The participants provided their written informed consent to participate in this study.

## Author contributions

ST: Conceptualization, Data curation, Funding acquisition, Methodology, Writing – original draft, Writing – review & editing. YJ: Conceptualization, Data curation, Formal analysis, Investigation, Methodology, Writing – original draft.
